# Supplementary material for: A Resilience Related Glial-Neurovascular Network Is Transcriptionally Activated after Chronic Social Defeat in Male Mice
Source: Cells. 2022 Oct 27;11(21):3405. doi: 10.3390/cells11213405 (PMC9655779; doi:10.3390/cells11213405)
Supplement: Supplementary file 1 [file cells-11-03405-s001.zip › cells-1899342-supplementary.pdf]

Supplementary Materials

Table S1. Number of cells per sample after filtering criteria step. From the 16 samples loaded on the 10X Genomics, after the filtering criteria, the left and right side were pooled. The DEGs analyses were performed on the dorsal and ventral parts of the hippocampus without distinction between the left and right side.

Group	Sample name	Number of cells kept after filtering	Number of cells in the pooled sample
control	left dorsal	1,814	3,646
	right dorsal	1,832	
	left ventral	1,773	3,624
	right ventral	1,851	
resilient	left dorsal	1,784	3,676
	right dorsal	1,892	
	left ventral	1,762	3,611
	right ventral	1,849	
intermediate	left dorsal	1,836	3,658
	right dorsal	1,822	
	left ventral	1,884	3,726
	right ventral	1,842	
susceptible	left dorsal	1,894	3,671
	right dorsal	1,777	
	left ventral	1,927	3,746
	right ventral	1,819	

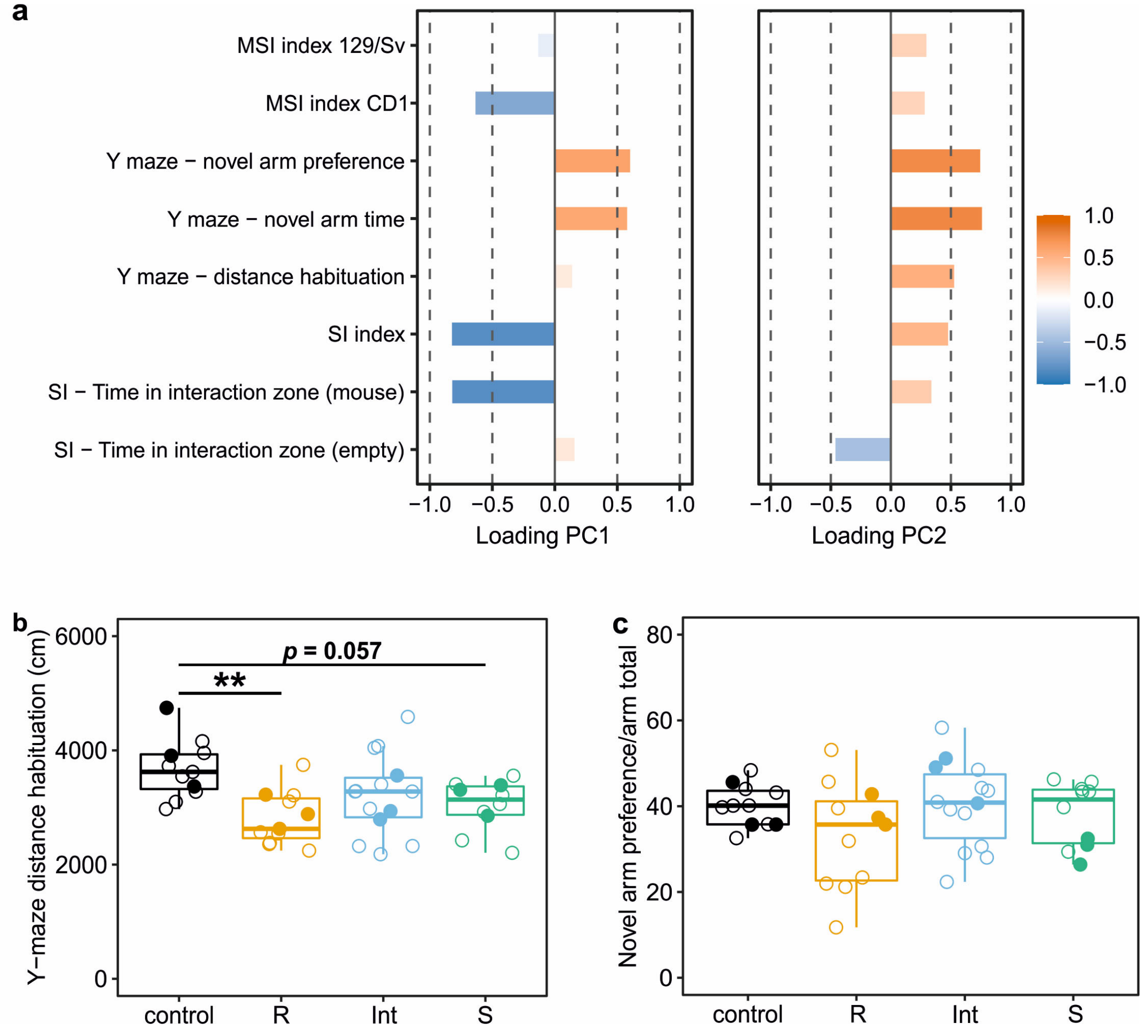

Supplementary Figure S1. Additional behavioural tests performed for elucidating phenotypes after CSDS. (**a**) Loadings of the behavioural outcome measures included in the PCA analysis on PC1 and PC2. **b-c.** Y-maze distance moved in the habituation phase (**b**) and novel arm preference out of total time (**c**) of the control, resilient (R), intermediate (Int) and susceptible (S) mice. Filled circles and triangles in **b-c**: individual mouse taken for subsequent single-cell RNA-seq experiment. $**p < 0.01$ (one-way ANOVA followed by Tukey's post hoc test in **b**).

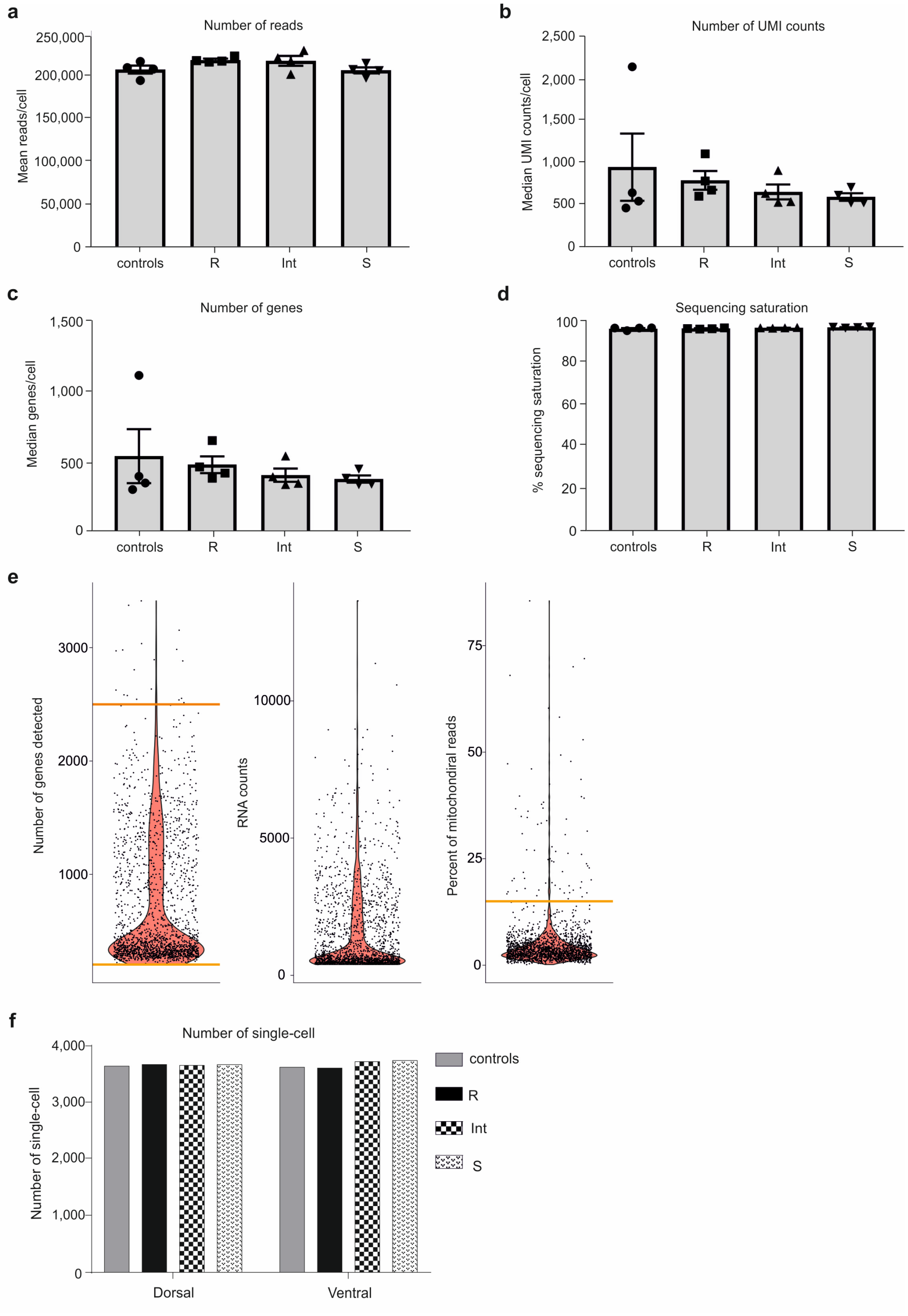

Supplementary Figure S2. Quality control of the single-cell RNA-seq experiment. (a) Number of reads for each sample within the different groups of mice: control, resilient (R), intermediate (Int), and susceptible (S) mice. (**b**) Number of Unique Molecular Identifiers (UMI) for each sample in each group. (**c**) Number of genes detected for each sample in each group. (**d**) Sequencing saturation for each sample in each group. (**e**) The number of genes, RNA counts, and percentage of mitochondrial reads detected in every single-cell within one sample. The orange lines represent the cut-off values: between 200 and 2,500 reads to exclude cell-free RNA or multiplets cells, <15% of mitochondrial gene reads to remove

any dead cells. (**f**) Total number of cells in each group of mice kept for the downstream analysis after the quality control in the hippocampus and in the dorsal, ventral, right, and left parts of the hippocampus.

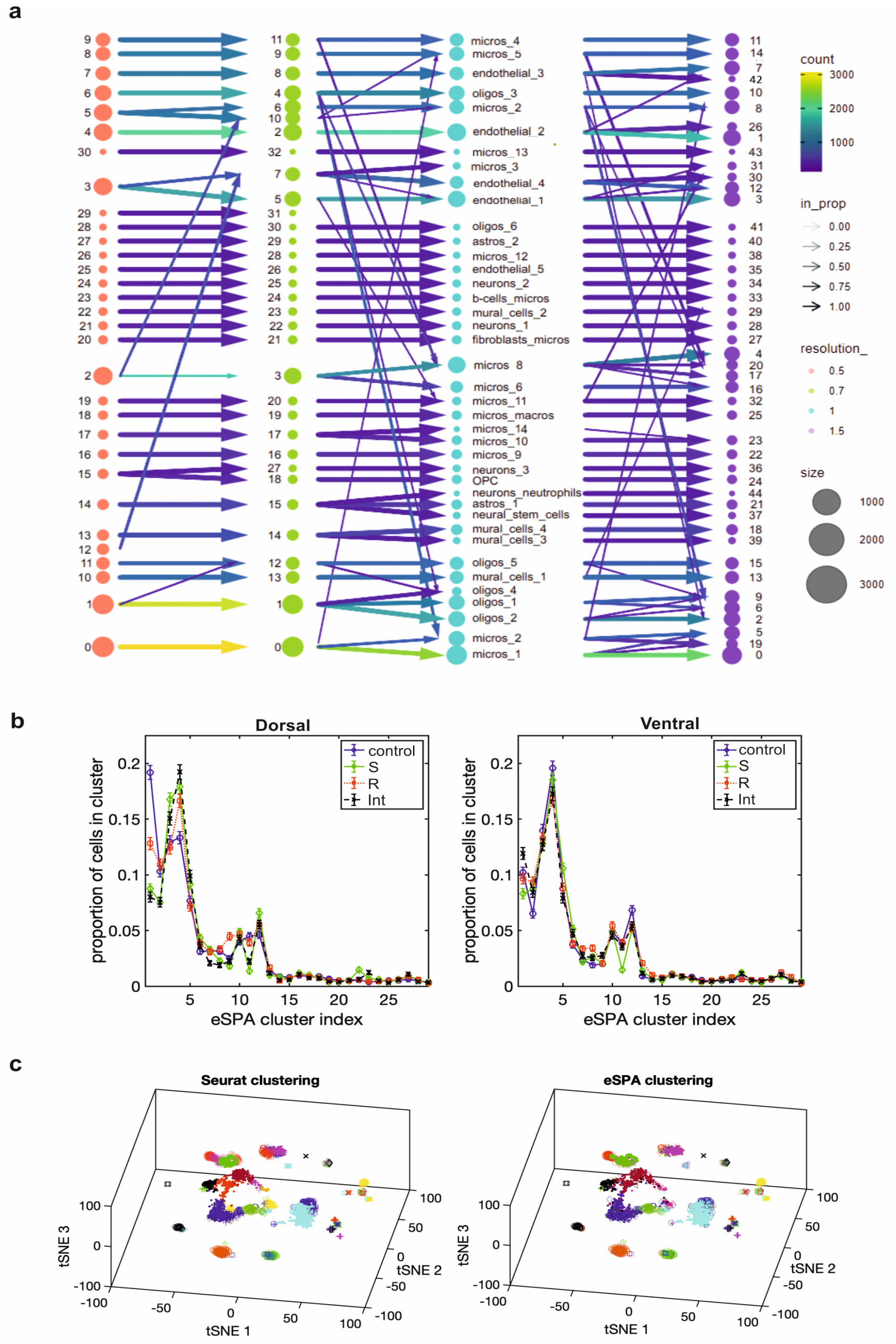

Supplementary Figure S3. Single-cell clustering parameter and eSPA analysis. (**a**) Clustree plot of different resolution parameter settings (0.5, 0.7, 1, 1.5), starting from top to bottom with the lowest value

to the highest value. Each circle represents a cluster of cells at the given resolution. The size of the cluster corresponds to the number of cells in the cluster. The arrows point to the clusters split in the next-higher resolution setting, and the transparency of the arrow is representative of the proportion of cells splitting up. Arrow colour corresponds to the number of cells following that particular split. Each cluster is labelled numerically after cluster size, or in case of resolution 1.0, after biologically relevant cell-type. (**b**) Results of the eSPA analysis for the dorsal, ventral, right and left hippocampal sub-regions for the control (blue lines, control), susceptible (green lines, S), resilient (red lines, R) and intermediate resilient (black lines, Int) groups. Bars indicate 95% binomial proportion confidence intervals from the robust Wilson score test. eSPA clustering reveals around 20 statistically significant clusters. Statistically significant between-group difference of the cell population proportions in the respective clusters is observed in the dorsal hippocampal sub-region. eSPA detects no statistically significant differences between the groups for the ventral hippocampal sub-region. (**c**) A t-SNE representation of the clustering results obtained with Seurat and eSPA. Results of the eSPA analysis for the considered single cell data reveal a robust cell clustering signatures and confirm our findings obtained with the Seurat analysis pipeline.

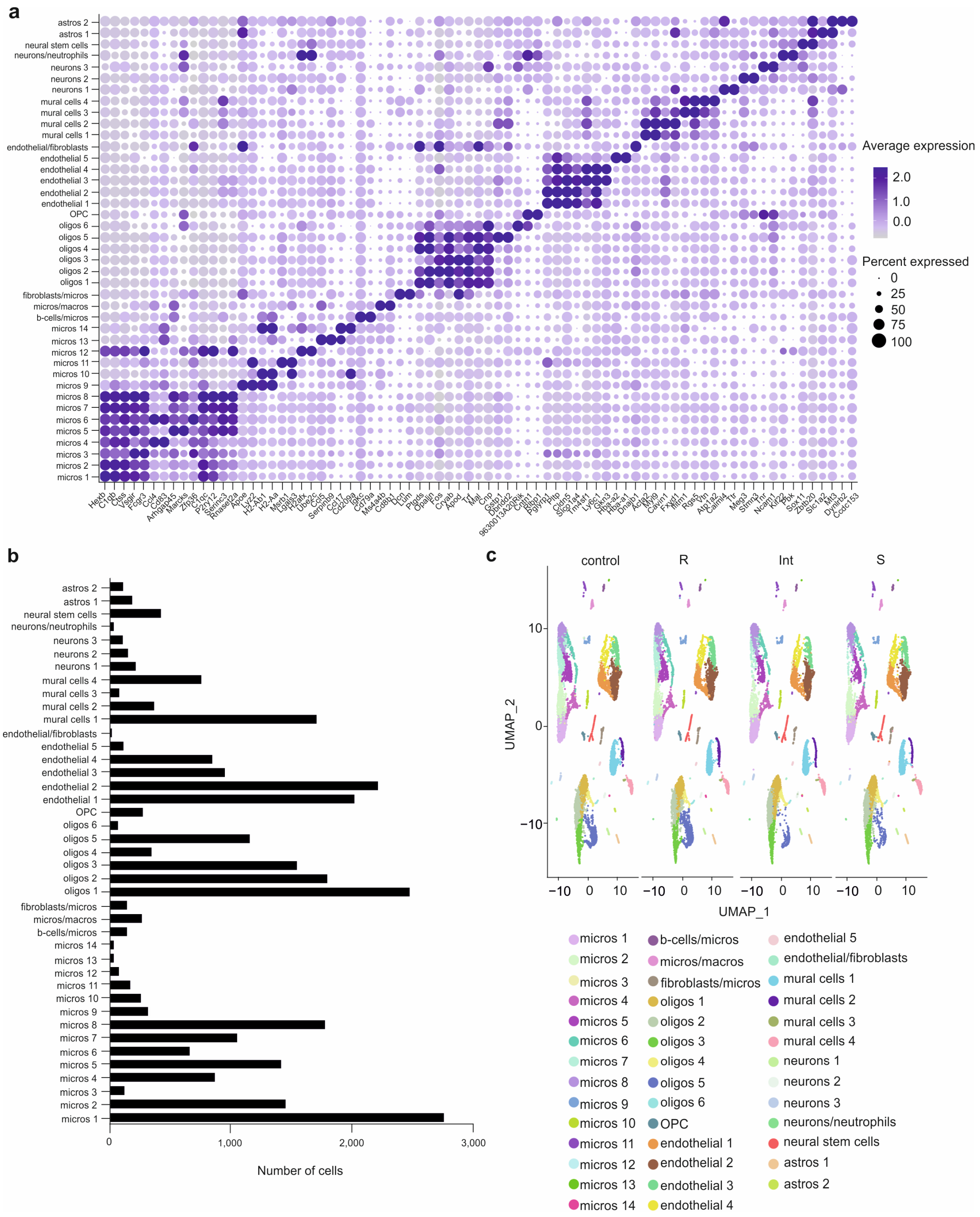

Supplementary Figure S4. Composition and repartition of the brain cell types among the clusters and the groups of mice. (**a**) Cell type annotation performed on the expression of TOP 2 genes per cluster. Dot plot representing the gene expression across all cell types. The size of the dot represents the percentage of cells expressing the gene, whereas the colour intensity of the dot corresponds to the average expression level. (**b**) Number of cells detected in all the 41 clusters. (**c**) UMAP visualization of the cell populations present in the hippocampus in control, resilient (R), intermediate (Int), and susceptible (S) mice.

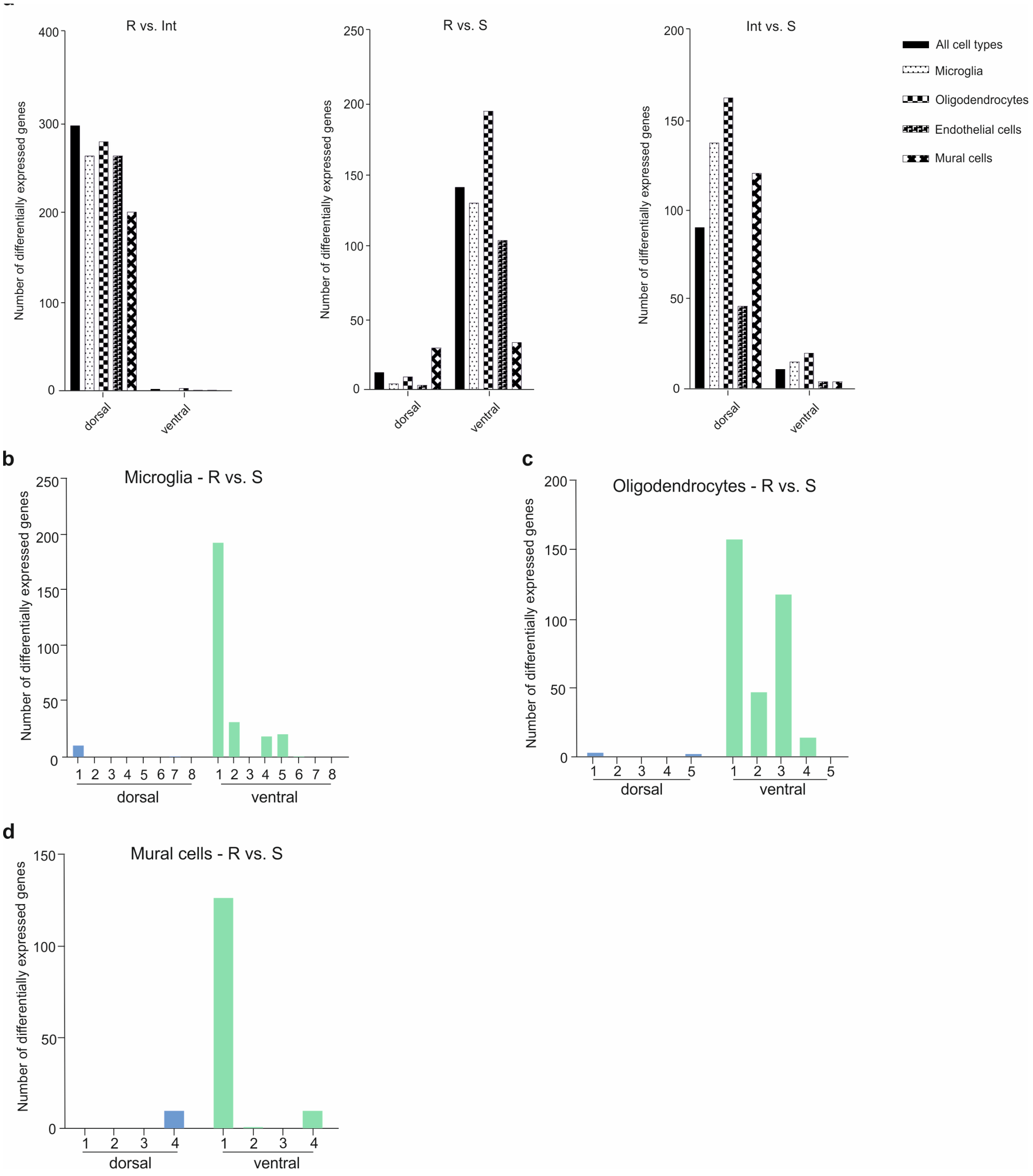

Supplementary Figure S5. **Differential expression analysis between the three groups of defeated animals: resilient, intermediate and susceptible**. (**a**) Number of differentially expressed genes between the three groups of defeated animals; i.e., resilient (R), intermediate (Int), and susceptible (S) mice, in each cell type. **b-d**. Number of DEGs in resilient mice (R) compared to the susceptible mice (S) in the microglia (**b**), oligodendrocytes (**c**) and mural cells (**d**) sub-clusters within the full hippocampus (hippocampus) and within the different parts: dorsal and ventral.

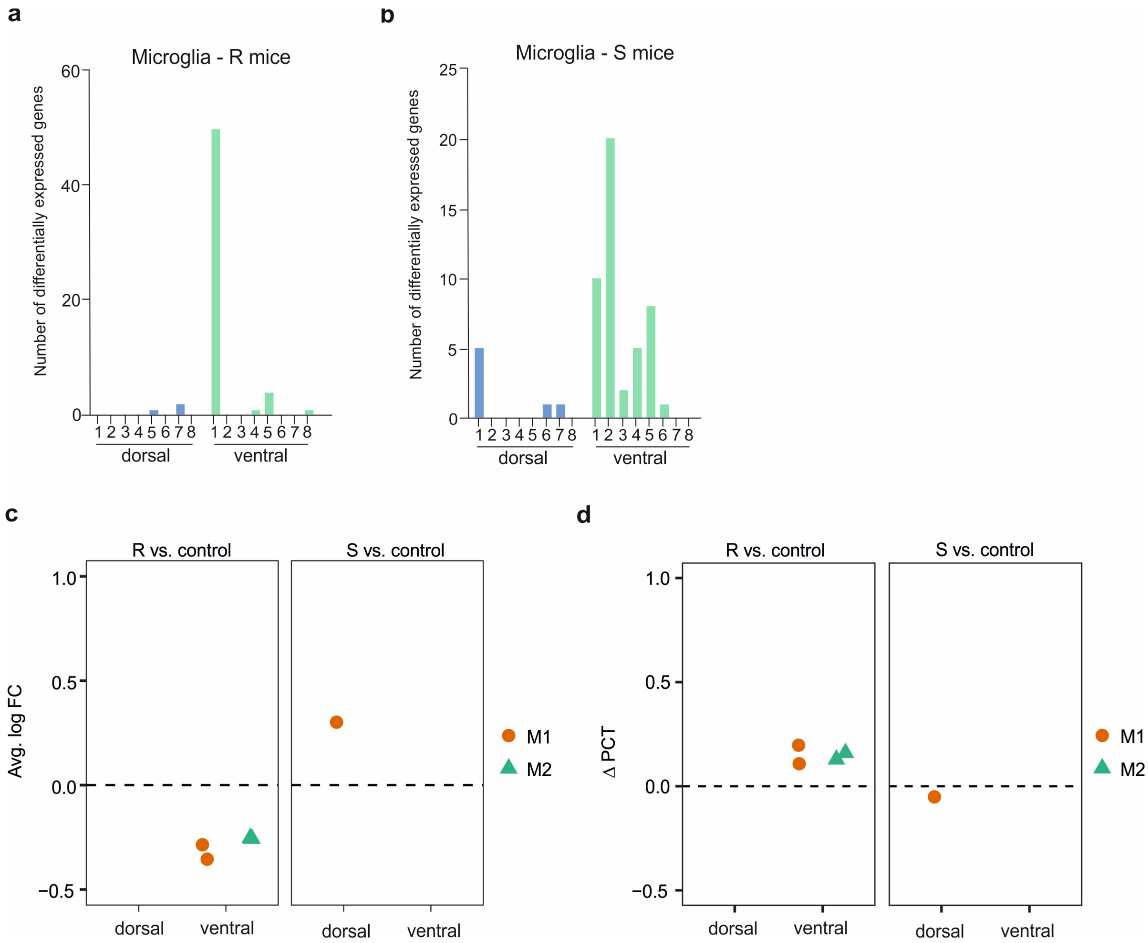

Supplementary Figure S6. Chronic social defeat stress response of microglial cells in the hippocampus of the resilient and susceptible mice. a-b. Number of DEGs among the microglial sub-clusters 1 to 8 in the resilient (R) (**a**), and in the susceptible (S) (**b**) mice compared to non-stressed controls in the entire hippocampus and in the dorsal and ventral, parts of the hippocampus. **c**. Differential expression of M1 (*Cxcl10, Fcgr2b, Fcgr3, H2-Aa, H2-D1, H2-Dmb1, H2-K1, H2-Oa, H2-Q4, H2-Q6, H2-T23, Tnfαip2, Tnfαip8l2, Il-1β*) and M2 (*Il-10rα, Socs3, Tgfβ1*) markers in the dorsal and ventral parts of the hippocampus in the resilient (R) and the susceptible (S) mice compared to non-stressed controls. (**d**) Difference in the percentage of cells (ΔPCT) expressing the M1 (*Cxcl10, Fcgr2b, Fcgr3, H2-Aa, H2-D1, H2-Dmb1, H2-K1, H2-Oa, H2-Q4, H2-Q6, H2-T23, Tnfαip2, Tnfαip8l2, Il-1β*) and M2 (*Il-10rα, Socs3, Tgfβ1*) markers in the dorsal and ventral parts of the hippocampus in the resilient (R) and the susceptible (S) mice compared to non-stressed controls.

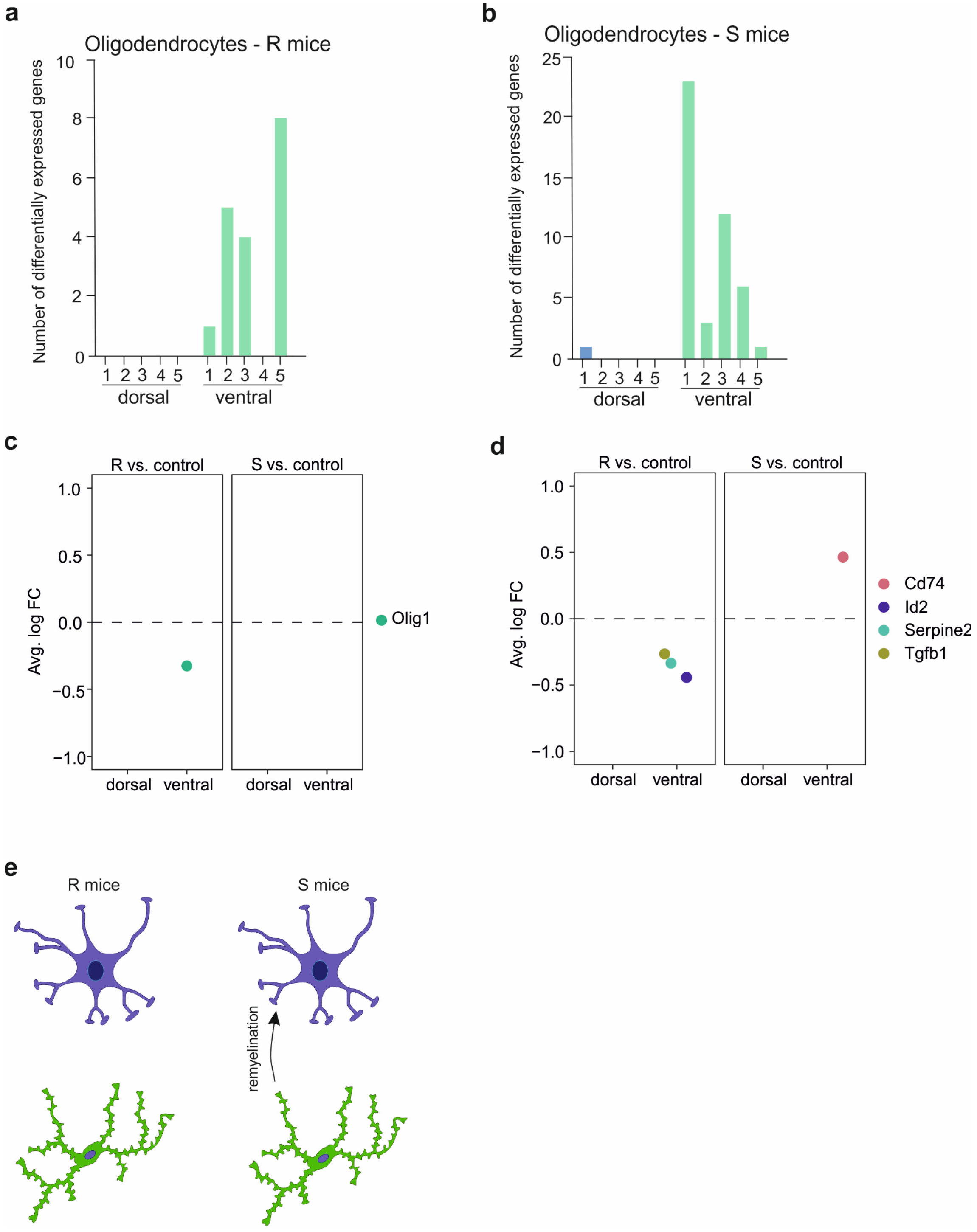

Supplementary Figure S7. Chronic social defeat stress response of the oligodendrocytes in the hippocampus of the resilient and susceptible mice. a-b. Number of DEGs among the oligodendrocyte sub-clusters 1 to 5 in the resilient (R) (**a**) and in the susceptible (S) (**b**) mice compared to non-stressed controls in the dorsal and ventral parts of the hippocampus. **c**. Differential expression of the oligodendrocyte progenitor (*Apod, H2-D1, Nkx6-2, Olig1, Sox8, Sox10*) in the resilient (R) and in the susceptible (S) mice compared to non-stressed controls in the dorsal and ventral parts of the hippocampus. (**d**) Differential expression of the genes involved in the positive regulation of glial cell differentiation pathway (*Cd74, Id2, Serpine2, Tgfβ1, H2-Aa, Lgals3, Lpl, Tnfαip2, Tnfαip28l2*) in the

microglia clusters in the resilient (R) and in the susceptible (S) mice compared to non-stressed controls in the dorsal and ventral parts of the hippocampus. **e**. Proposed cell-cell interaction identified in the R and S mice between oligodendrocytes (upper panel) and microglia (lower panel).

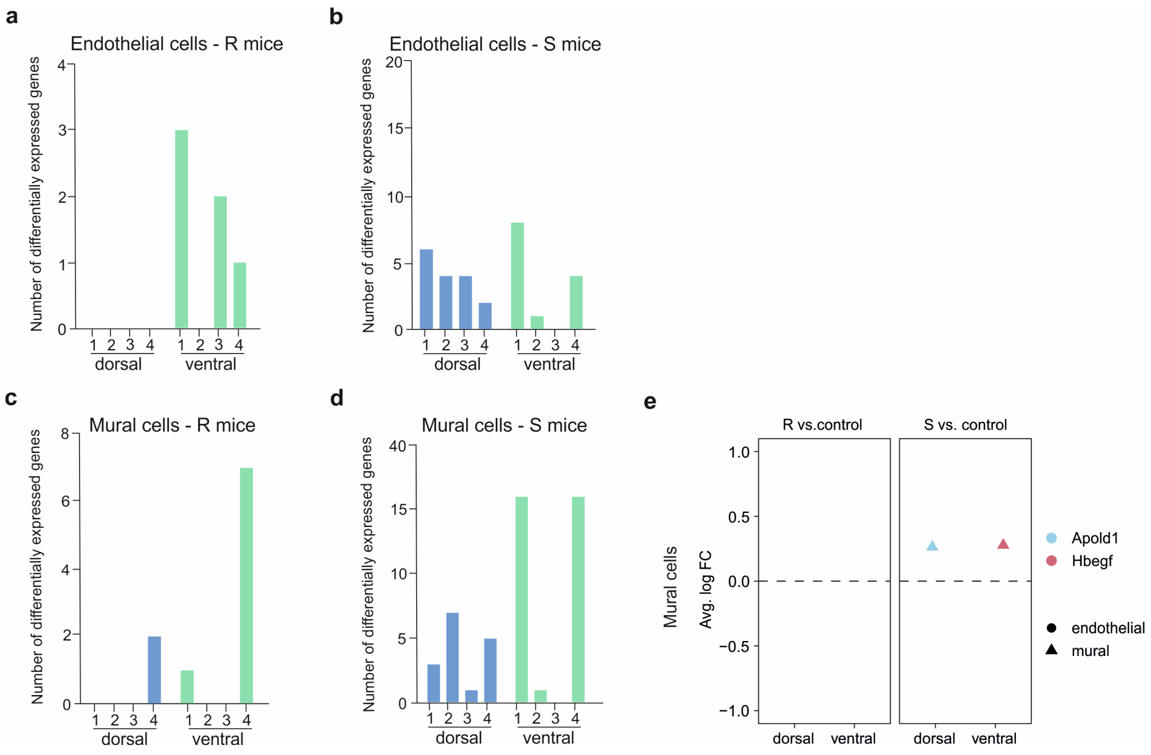

Supplementary Figure S8. Involvement of the hippocampus' endothelial and mural cells after chronic social defeat stress of the resilient and susceptible mice. a-b. Number of DEGs among the endothelial sub-clusters 1 to 4 in the resilient (R) (**a**) and in the susceptible (S) (**b**) mice compared to non-stressed controls in the dorsal and ventral parts of the hippocampus. **c-d**. Number of DEGs among the mural sub-clusters 1 to 4 in the resilient (R) (**c**) and susceptible (S) (**d**) mice compared to non-stressed controls in the dorsal and ventral parts of the hippocampus. (**e**) Differential expression of the genes involved in the angiogenesis pathways in the endothelial and mural cells of resilient and susceptible mice compared to non-stressed controls in the dorsal and ventral parts of the hippocampus.

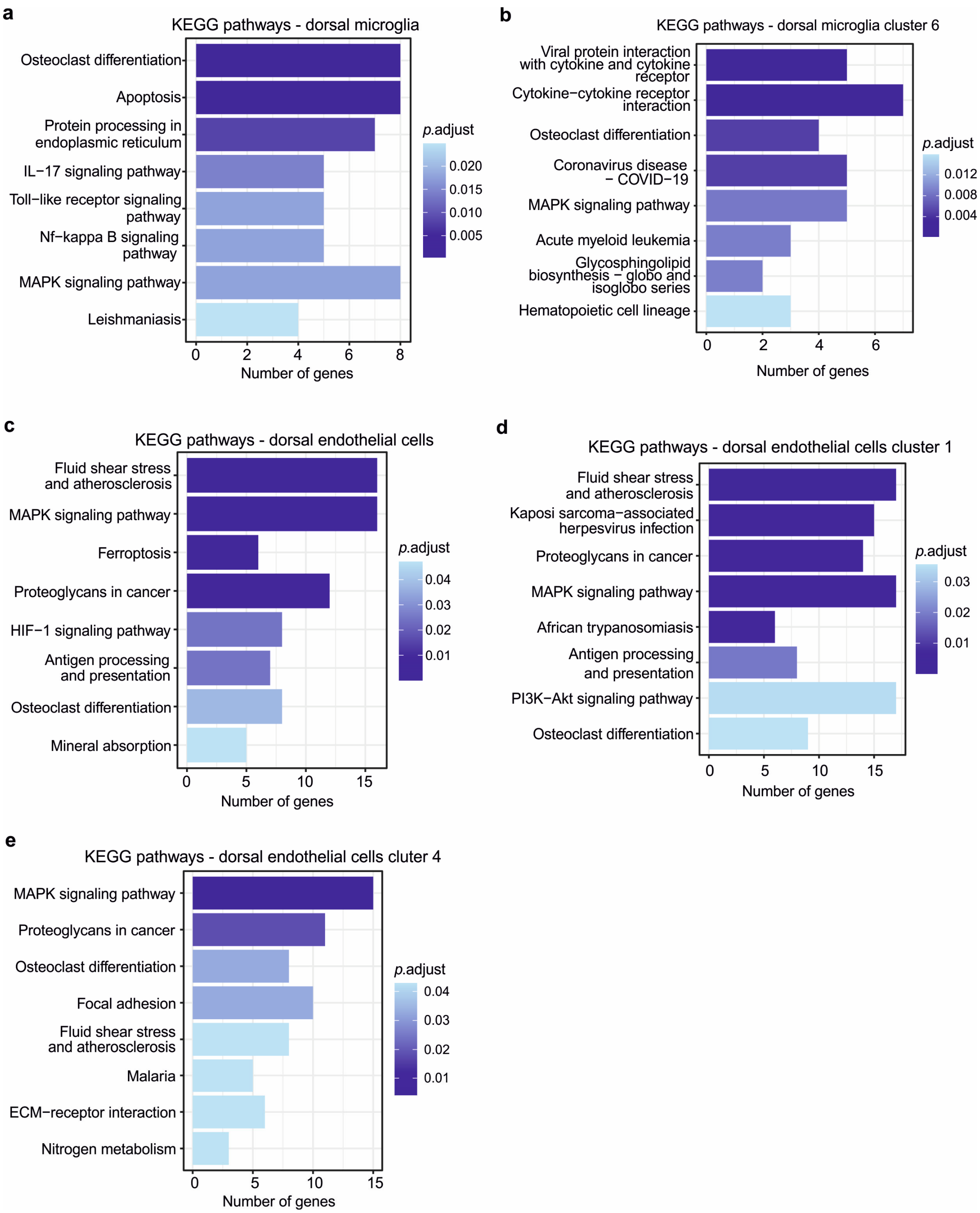

Supplementary Figure S9. Negative regulation of mTOR in the dorsal hippocampus of Int mice. KEGG pathway analysis revealed that the negative mTOR regulator pathways PI3K-Akt and MAPK are upregulated in the microglia (**a**, **b**) and in the endothelial cells (**c**-**e**) of the dorsal hippocampus of Int mice.
